# Supplementary material for: Controlled Reduction of Graphene Oxide Using Sulfuric Acid
Source: Materials (Basel). 2020 Dec 25;14(1):59. doi: 10.3390/ma14010059 (PMC7795585; doi:10.3390/ma14010059)
Supplement: Supplementary file 1 [file materials-14-00059-s001.pdf]

Supporting Information

Controlled Reduction of Graphene Oxide Using Sulfuric Acid

Ana Cecilia Reynosa-Martínez [1], Erika Gómez-Chayres [1], Rafael Villaurrutia [2], Eddie López-Honorato [1,3,*]

[1] Centro de Investigación y de Estudios Avanzados del IPN, Unidad Saltillo, Av. Industria Metalúrgica 1062, Parque Industrial, Ramos Arizpe 25900, Coahuila, Mexico; cecilia.reynosa@cinvestav.edu.mx (A.C.R.-M.); erika.chayres@cinvestav.mx (E.G.-C.)

[2] Thermo Fisher Scientific de México, Avenida Morones Prieto 2805 Pte., Monterrey 64710, Nuevo León, Mexico; rafael.arenas@thermofisher.com

[3] Oak Ridge National Laboratory, Oak Ridge, TN 37831, USA

* Correspondence: honoratole@ornl.gov

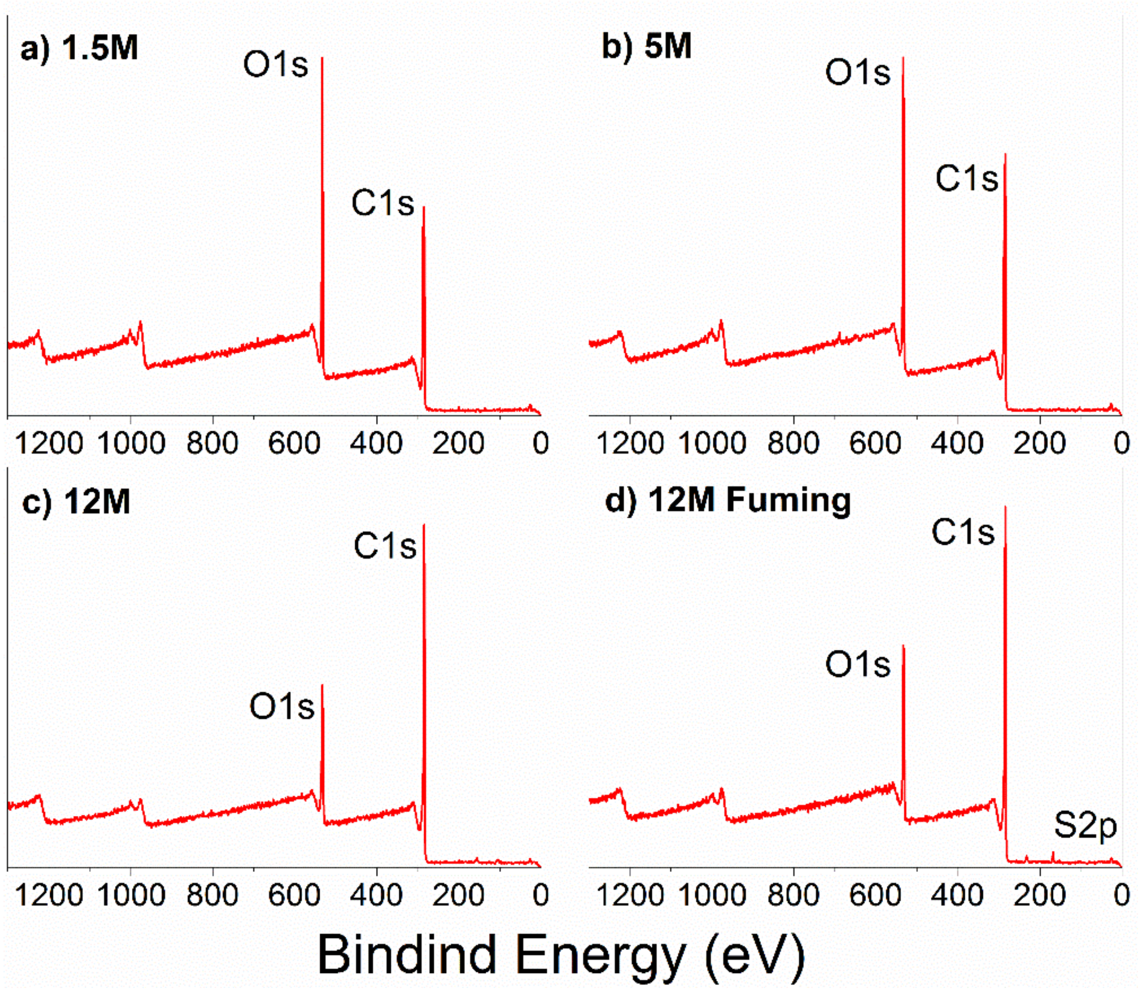

Figure S1. XPS narrow scan of RGO treated with H_2SO_4 at a) 1.5 M, b) 5M, and c) 12 M and fuming H_2SO_4 at d) 12 M. The presence of sulfur it can be observed only in the RGO treated with fuming H_2SO_4 at 12 M, due to the excess of sulfur trioxide (SO_3) in the acid.

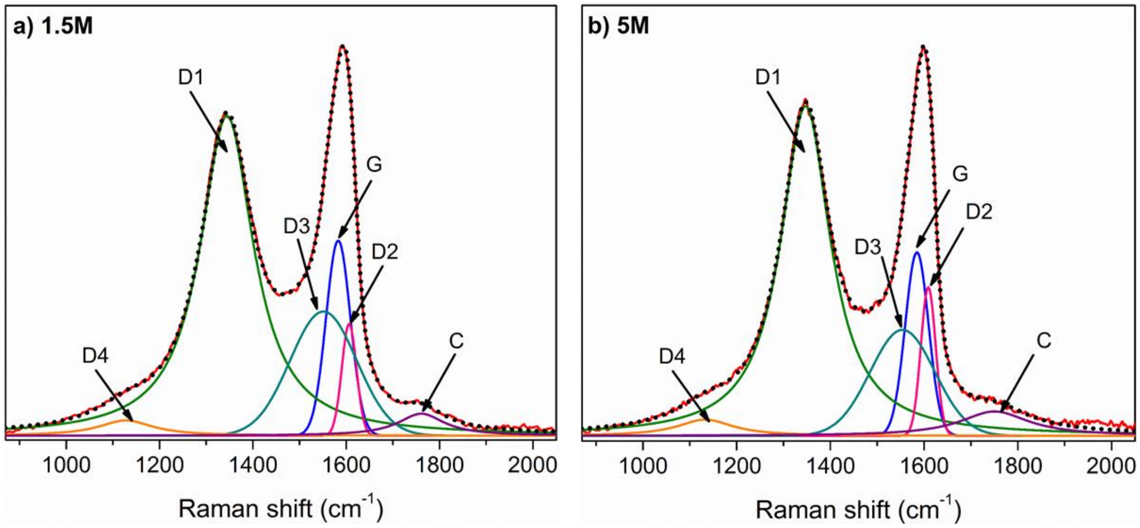

Figure S2. Raman spectroscopy spectra of GO treated with H_2SO_4 in (a) 1.5 M and (b) 5 M concentrations.

Table S1. Position of bands G and D in Raman spectra and I_D/I_G ratio for as-produced GO; GO treated with H_2SO_4 in concentrations of 1.5, 5, and 12 M; GO treated with fuming H_2SO_4 in concentration of 12 M; and OG irradiated at 74 μW/cm^2 for 120 h.

	**G**	**±**	**D**	**±**	**I_D/I_G**	**±**
**OG**	1,591.83	3.28	1,356.04	0.57	1.62	0.17
**1.5 M**	1,581.77	1.46	1,344.89	0.40	1.66	0.10
**5 M**	1,584.52	1.97	1,346.39	0.63	1.75	0.32
**12 M**	1,599.28	2.15	1,357.97	0.61	1.57	0.03
**Fuming 12 M**	1,599.28	1.70	1,356.04	1.02	1.45	0.13
**74 μW/cm^2 120 h**	1,593.78	1.93	1,357.30	0.46	1.83	0.11

Table S2. Position of bands D2, D3, D4, and C in Raman spectra for as-produced GO; GO treated with H_2SO_4 in concentrations of 1.5, 5, and 12 M; GO treated with fuming H_2SO_4 in concentration of 12 M; and OG irradiated at 74 μW/cm^2 for 120 h.

	**D2**	**±**	**D3**	**±**	**D4**	**±**	**C**	**±**
**OG**	1,615.99	1.25	1,562.75	3.06	1,141.75	4.02	1,769.99	3.83
**1.5 M**	1,605.24	0.83	1,548.91	1.46	1,127.07	3.59	1,759.56	3.87
**5 M**	1,608.78	1.60	1,553.09	3.05	1,128.94	3.19	1,752.25	2.61
**12 M**	1,621.34	1.68	1,566.05	3.31	1,138.30	2.82	1,763.58	2.87
**Fuming 12 M**	1,621.34	1.83	1,566.05	2.81	1,138.31	9.51	1,763.58	5.02
**74 μW/cm^2 120 h**	1,616.49	1.16	1,558.95	2.19	1,142.90	1.63	1,750.10	2.82

Table S3. Values for the FWHM of bands G and D in Raman spectra for as-produced GO, GO treated with H_2SO_4 in concentrations of 1.5, 5, and 12 M; GO treated with fuming H_2SO_4 in concentration of 12 M; and GO irradiated at 74 μW/cm^2 for 120 h.

	**G FWHM**	**±**	**D FWHM**	**±**
**OG**	57.92	1.20	128.16	8.58
**1.5 M**	61.98	1.46	136.14	1.28
**5 M**	58.00	1.97	130.51	2.41
**12 M**	54.12	2.15	135.16	2.05
**Fuming 12 M**	54.07	1.70	135.16	5.29
**74 μW/cm^2 120 h**	61.88	1.93	148.91	4.78

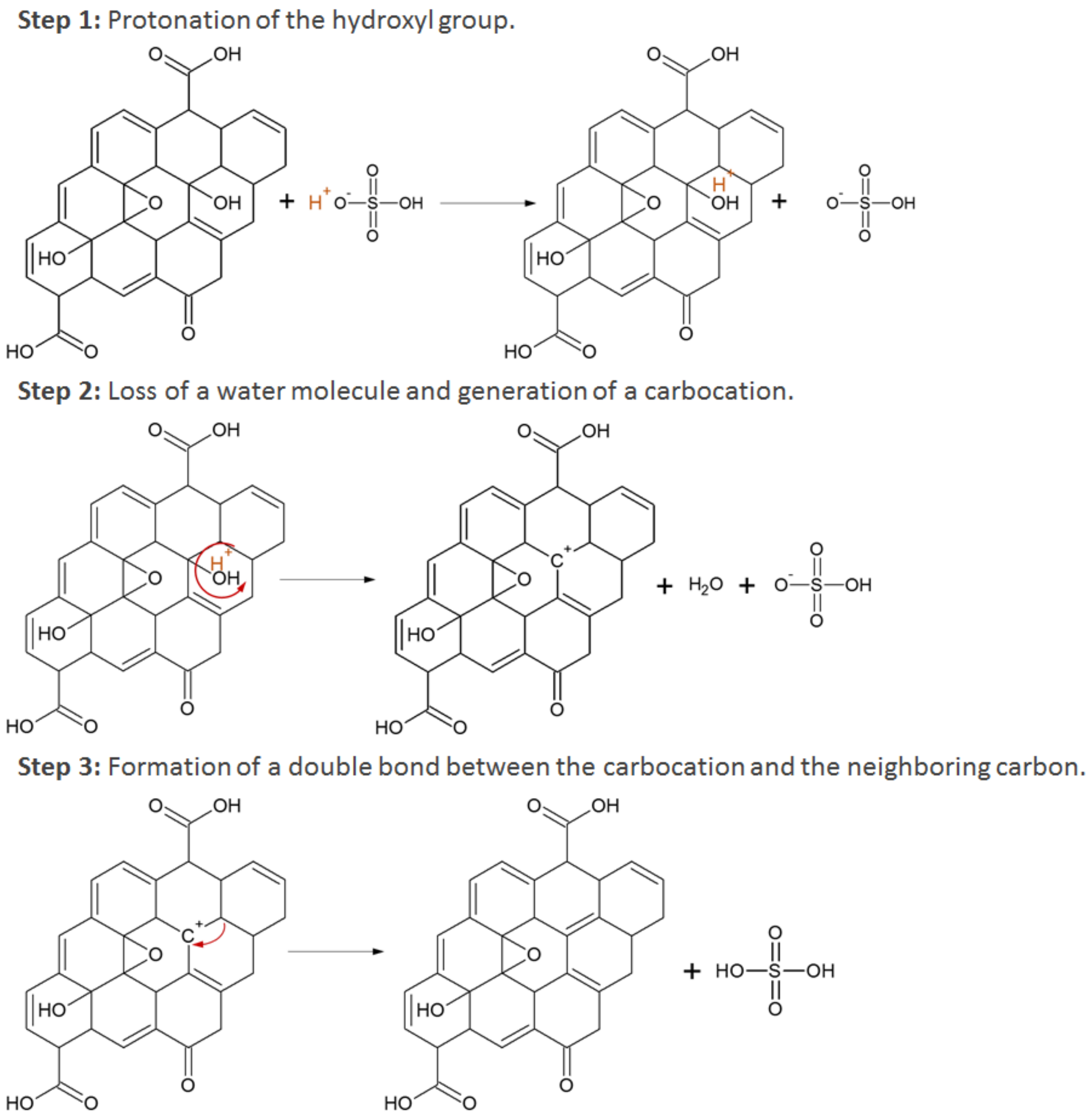

Figure S3. Reduction mechanism of GO by the loss of -OH functional group by the effect of H_2SO_4.

Step 1: Protonation of the epoxide group.

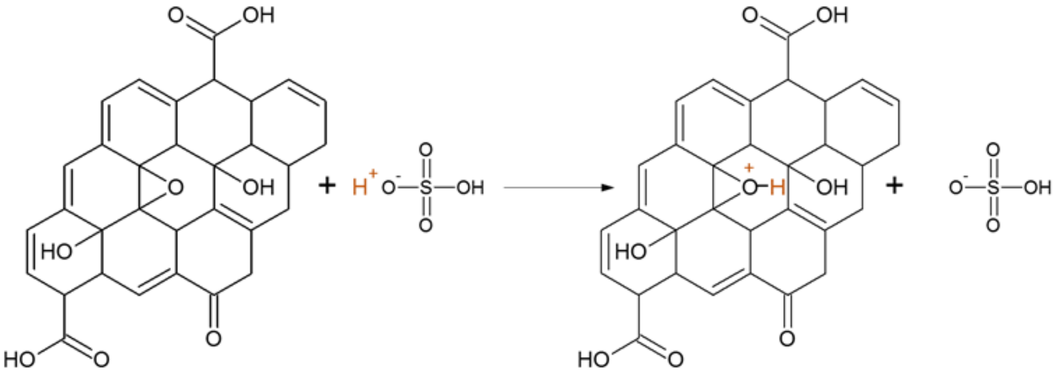

Step 2: Nucleophilic attack of the water towards one of the carbons.

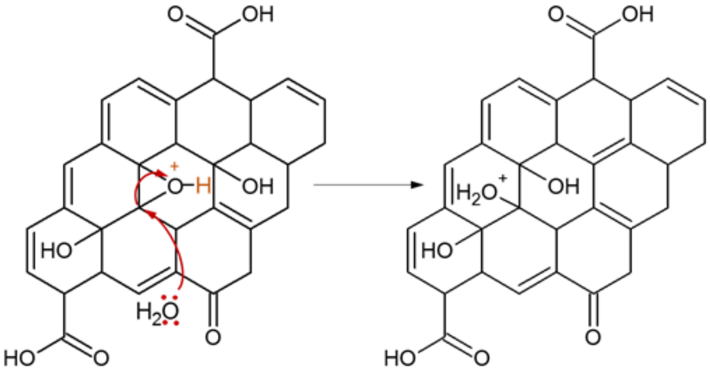

Step 3: Deprotonation and formation of hydroxyl groups.

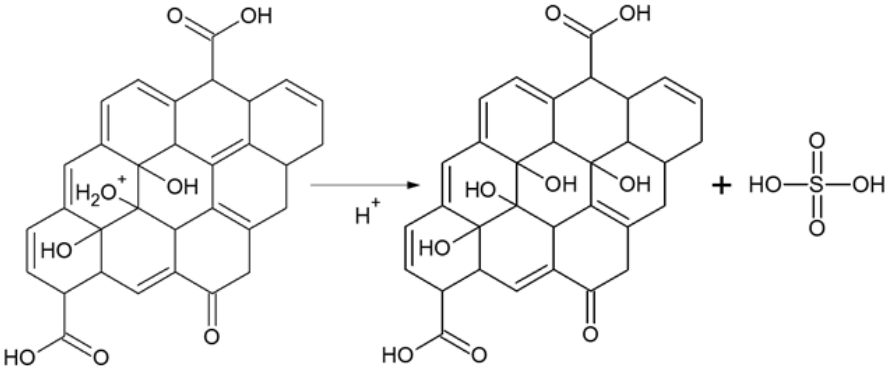

Figure S4. Reduction mechanism of GO by the opening of C-O-C functional group (de-epoxidation) by the effect of H_2SO_4.
